# Simulated Tissue Contouring Using an Ovate Pontic Design: A Detailed Case Report

**DOI:** 10.7759/cureus.80276

**Published:** 2025-03-09

**Authors:** Sumeet Agarwal, Uttam Shetty, Laresh N Mistry, Prasad Mhaske, Shantanu Deshpande, Saba Kondkari, Sayem A Mulla, Himmat Jaiswal, Hrashikesh Vaidya

**Affiliations:** 1 Prosthodontics, Bharati Vidyapeeth (Deemed to be University) Dental College and Hospital, Navi Mumbai, IND; 2 Pediatric and Preventive Dentistry, Bharati Vidyapeeth (Deemed to be University) Dental College and Hospital, Navi Mumbai, IND; 3 Dentistry, Bharati Vidyapeeth (Deemed to be University) Dental College and Hospital, Navi Mumbai, IND; 4 Conservative Dentistry and Endodontics, Bharati Vidyapeeth (Deemed to be University) Dental College and Hospital, Navi Mumbai, IND

**Keywords:** aesthetic dentistry, anterior restoration, case report, cosmetic dentistry, missing teeth, ovate

## Abstract

This case study investigates the use of an ovate pontic design in simulated tissue contouring, a technique intended to produce the best possible functional and aesthetic results in dental prostheses. The edentulous ridge and normal gingival architecture must be preserved and maintained, as they frequently collapse after tooth extraction. In today's aesthetic dentistry, preserving interproximal tissue contour and preventing alveolar bone collapse are highly desirable.

This case report presents a 25-year-old male patient with a missing anterior tooth due to trauma. The patient's missing anterior maxillary tooth caused both functional and aesthetic problems. The patient was presented with two options: an implant-supported prosthesis or a three-unit fixed partial denture (FPD). Due to economic constraints, the patient opted for a three-unit FPD. A clinical examination revealed that the adjacent teeth were intact and that the edentulous ridge was well-maintained. This necessitated a careful prosthetic approach to achieve harmonious integration with the surrounding soft tissues. A transfer impression technique was used to attain the desired result. A three-unit FPD was selected to restore the patient's edentulous area and provide an anatomical imitation of normal gingival architecture, incorporating an ovate pontic and porcelain-fused-to-metal crowns on both adjacent teeth. Because of its natural appearance and improved integration with the edentulous ridge, the ovate pontic design is preferable to other pontic designs, such as the modified ridge lap or the saddle pontic. Compared to bulkier or ill-fitting designs, its shape closely resembles the contour of a natural tooth, improving cleanliness and aesthetics by reducing plaque retention. The results demonstrated that dental aesthetics and functional occlusion could be successfully restored, and the patient was satisfied with the prosthesis's natural-looking appearance.

This case highlights the crucial role of custom prosthetic methods in creating natural-looking dental restorations, particularly through ovate pontic design and simulated tissue contouring. It underscores the importance of combining patient-centered care with cutting-edge prosthodontic concepts such as digital technology, 3D printing, and artificial intelligence to optimize outcomes in dental practice. From an educational perspective, this paper provides valuable insights into how a three-unit FPD with an ovate pontic enhances the functional and aesthetic aspects of dental prostheses.

## Introduction

With increased patient aesthetic expectations, dentists face the challenging task of replacing missing teeth with maximum aesthetics while maintaining overall function and health. When teeth are unrestorable, clinicians must utilize various techniques to preserve or restore both function and aesthetics. The edentulous ridge and normal gingival architecture must be preserved and maintained, as they often collapse after tooth extraction.

The presence or absence of teeth determines the presence or absence of the interdental papilla. When papillae are absent from edentulous ridges, the situation is referred to as "no teeth, no papillae." Consequently, following extraction, it is essential to achieve the gingival contour of the natural teeth through proper tooth contact and pontic design, creating the illusion of a tooth emerging from the gingiva, as well as the necessary emergence profile to achieve "pink aesthetics" and eliminate black triangles. After an anterior tooth extraction, the post-extraction socket must be maintained in the same position and form as before the extraction to prevent interproximal tissue recession. The ovate pontic is one such method for achieving this form.

When the gingival contour of the tooth remains intact following a recent extraction, such as in cases where root piece extraction is advised for an anterior tooth, an ovate pontic is indicated. However, patients with poor oral hygiene and a narrow, knife-edge ridge (Siebert's ridge defect) are contraindicated for the ovate pontic procedure. Due to its natural appearance and improved integration with the edentulous ridge, the ovate pontic design is sometimes preferable to other pontic designs, such as the modified ridge lap or the saddle pontic. Compared to bulkier or ill-fitting designs, its shape closely resembles the contour of a natural tooth, improving cleanliness and aesthetics by reducing plaque retention.

Advantages of the ovate pontic include a better emergence profile and improved aesthetics. Additionally, flossing is easier than with a modified ridge lap pontic. It also provides a more effective speech air seal compared to a modified ridge lap pontic. When properly designed, the ovate pontic enhances functionality by preventing the passage of air or saliva while reducing the appearance of black triangles, thereby creating the illusion of a gingival border and papilla.

## Case presentation

**Figure 1:**
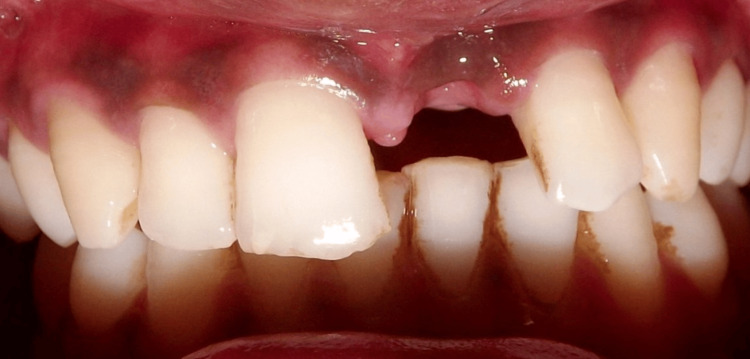
Pre-treatment

A 25-year-old male patient visited the Department of Prosthodontics and Crown and Bridge with the chief complaint of restoring the lost crown structure due to trauma in the upper front area (Figure 1). Figure 1 shows the pre-treatment image of the patient's anterior crown loss due to trauma.

Upon further examination, the patient provided a history of a fall 20 days ago, which resulted in a traumatic injury to his anterior teeth. The patient, being young and employed as a front desk manager, was inclined toward achieving the best esthetic results but had economic constraints. Radiographic examination revealed periapical lesions associated with teeth 11, 21, and 22, rendering them non-vital. A detailed clinical examination showed grade 1 mobility in teeth 11 and 22, a submerged root stump concerning tooth 21 (the primary tooth of concern), an incisal fracture in teeth 11 and 22, and a high smile line.

The patient was presented with the options of an implant-supported prosthesis, a fixed partial denture (FPD), and a removable partial denture (RPD). Due to the patient's financial constraints and lack of interest, an implant-supported prosthesis was excluded. RPD was ruled out due to the patient's non-compliance. Considering the patient’s demand for maximum esthetics and economic limitations, a three-unit FPD using porcelain-fused-to-metal with an ovate pontic design was planned, preceded by immediate temporization. The patient was informed about the prolonged treatment duration due to the selected pontic type, the periodic visits required, and the necessary maintenance. The patient agreed to the proposed treatment plan.

**Figure 2:**
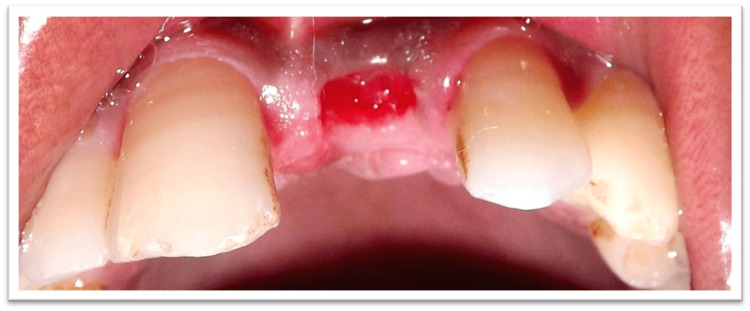
Socket after extraction of root piece

Tooth number 21's root piece was deemed non-restorable and indicated for extraction (Figure 2), followed by an immediate temporary restoration. After the extraction of the root piece of the concerned tooth, a provisional restoration was planned and used as a template to preserve the tissue contour. Figure 2 shows the post-extraction socket of tooth 21.

**Figure 3:**
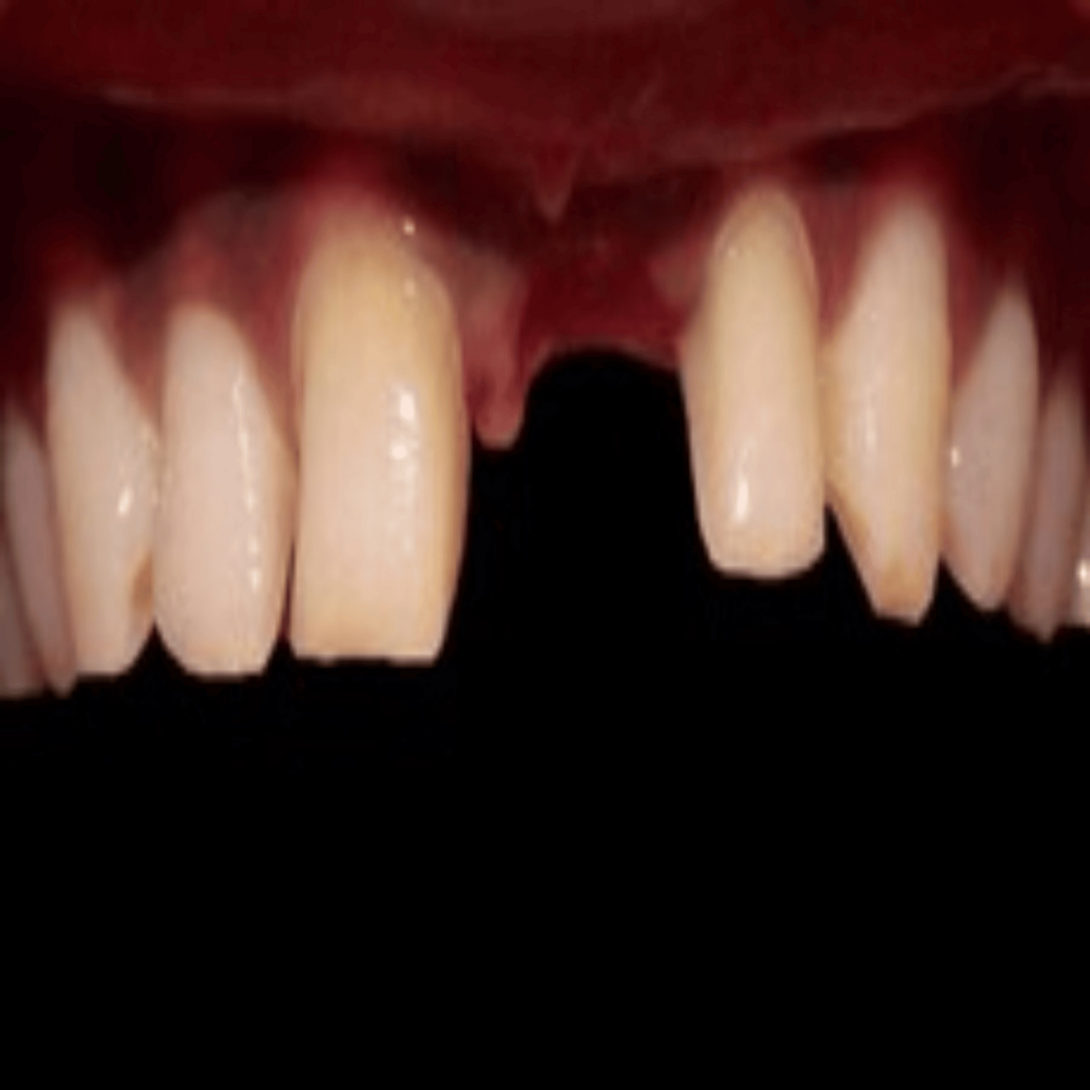
Tooth preparation after root canal treatment

**Figure 4:**
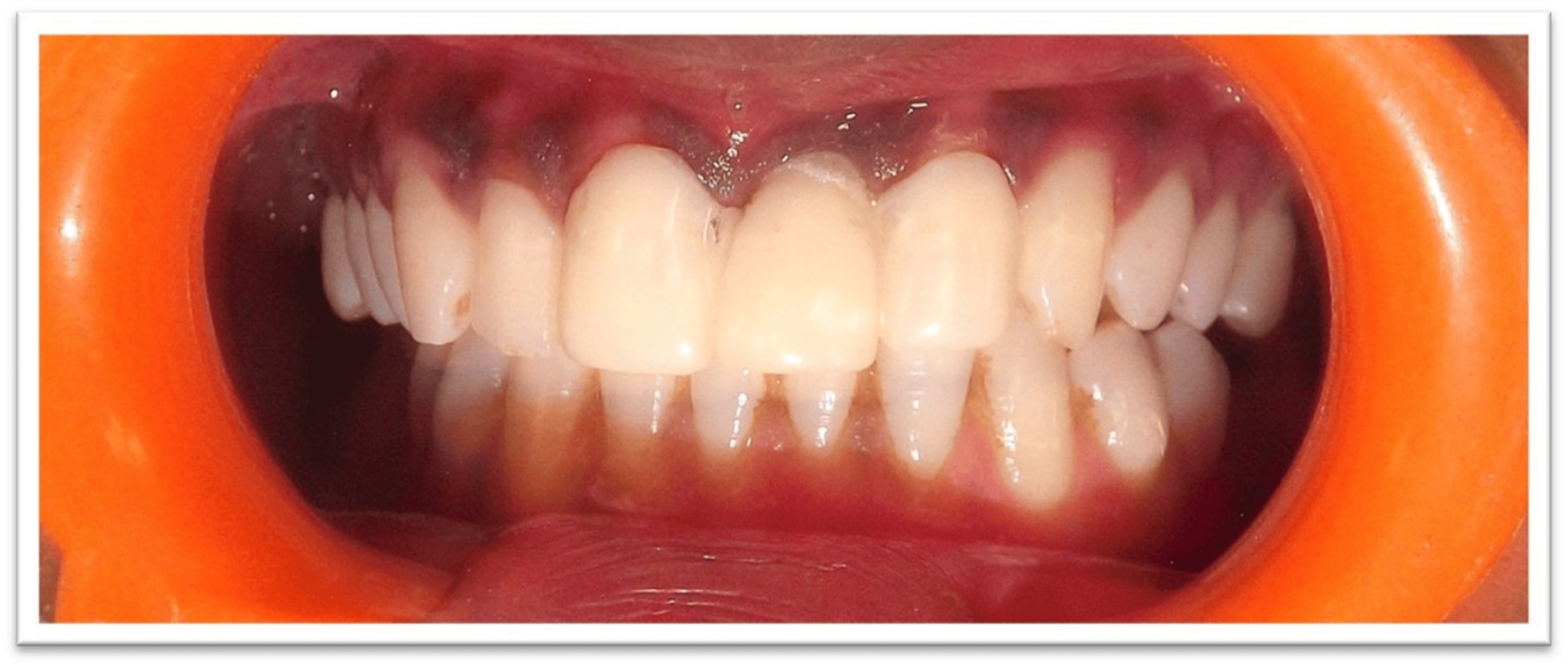
Temporization

Here, the temporary restoration also serves as a splint for the mobile abutments. Therefore, a shallow chamfer margin was prepared (Figure 3) to accommodate a temporary restoration (Figure 4) without any premature contacts. Figure 3 illustrates the tooth preparation for tooth numbers 11 and 22, while Figure 4 depicts the placement of the temporary restoration.

Evaluation phase

**Figure 5:**
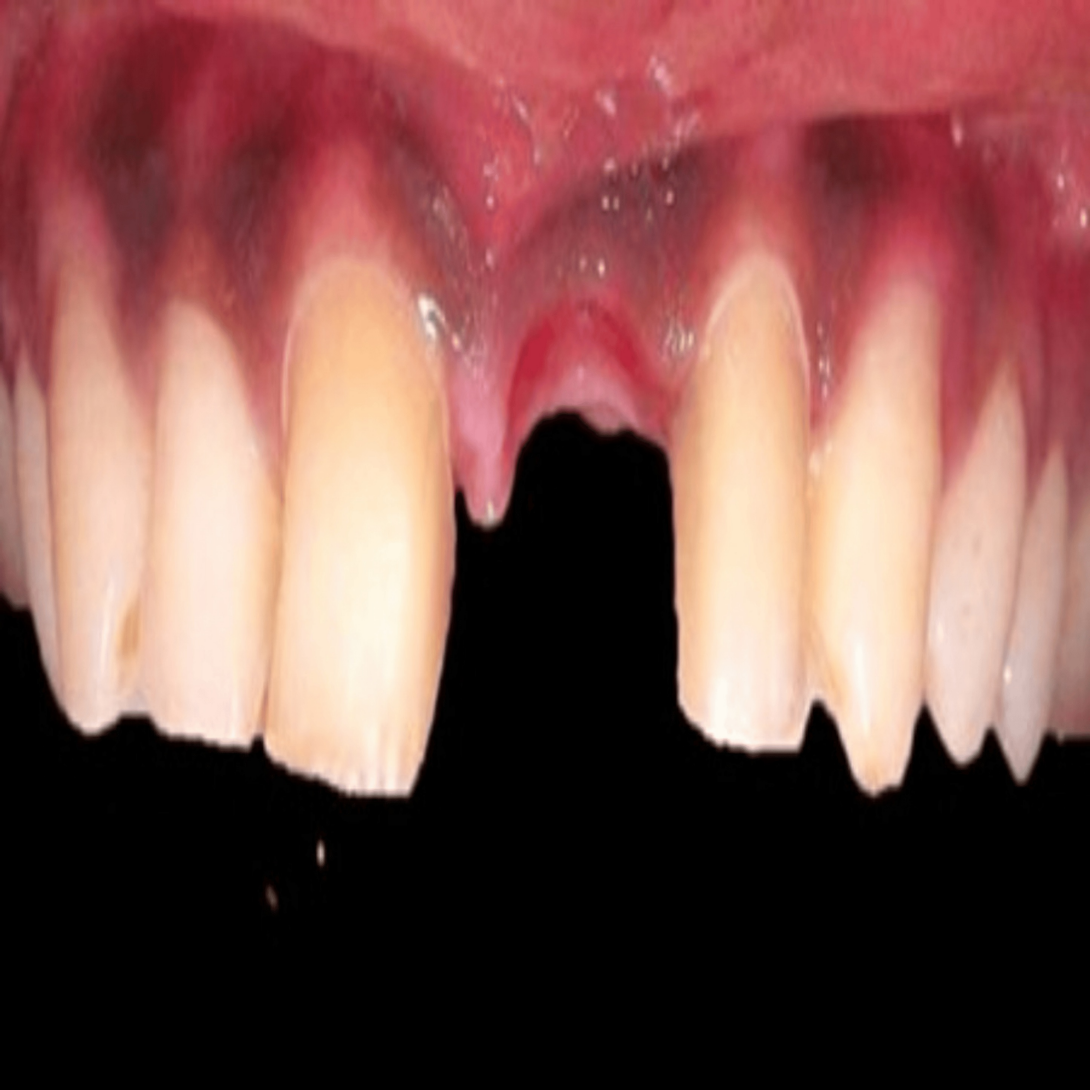
Evaluation after one week

**Figure 6:**
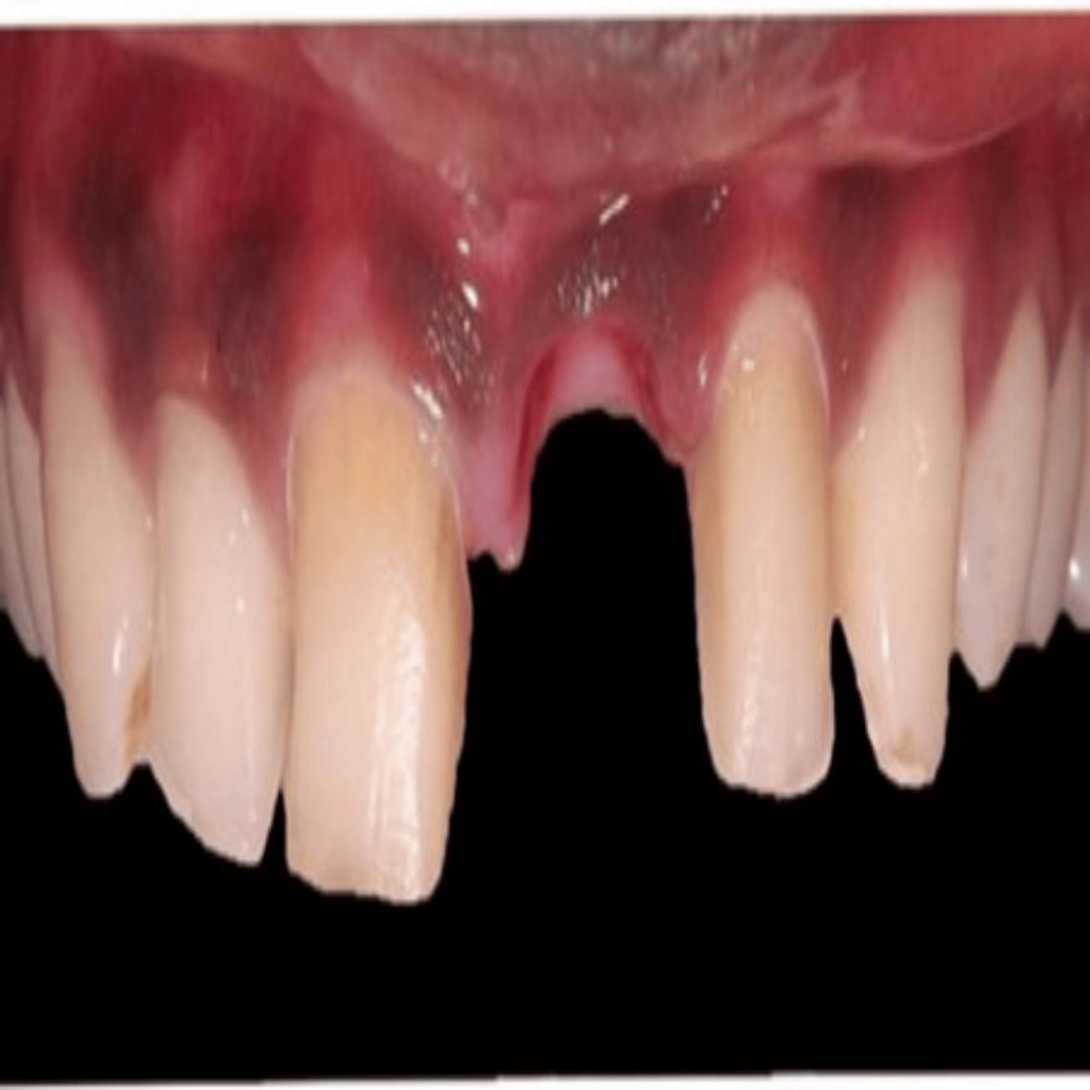
Evaluation after two weeks

**Figure 7:**
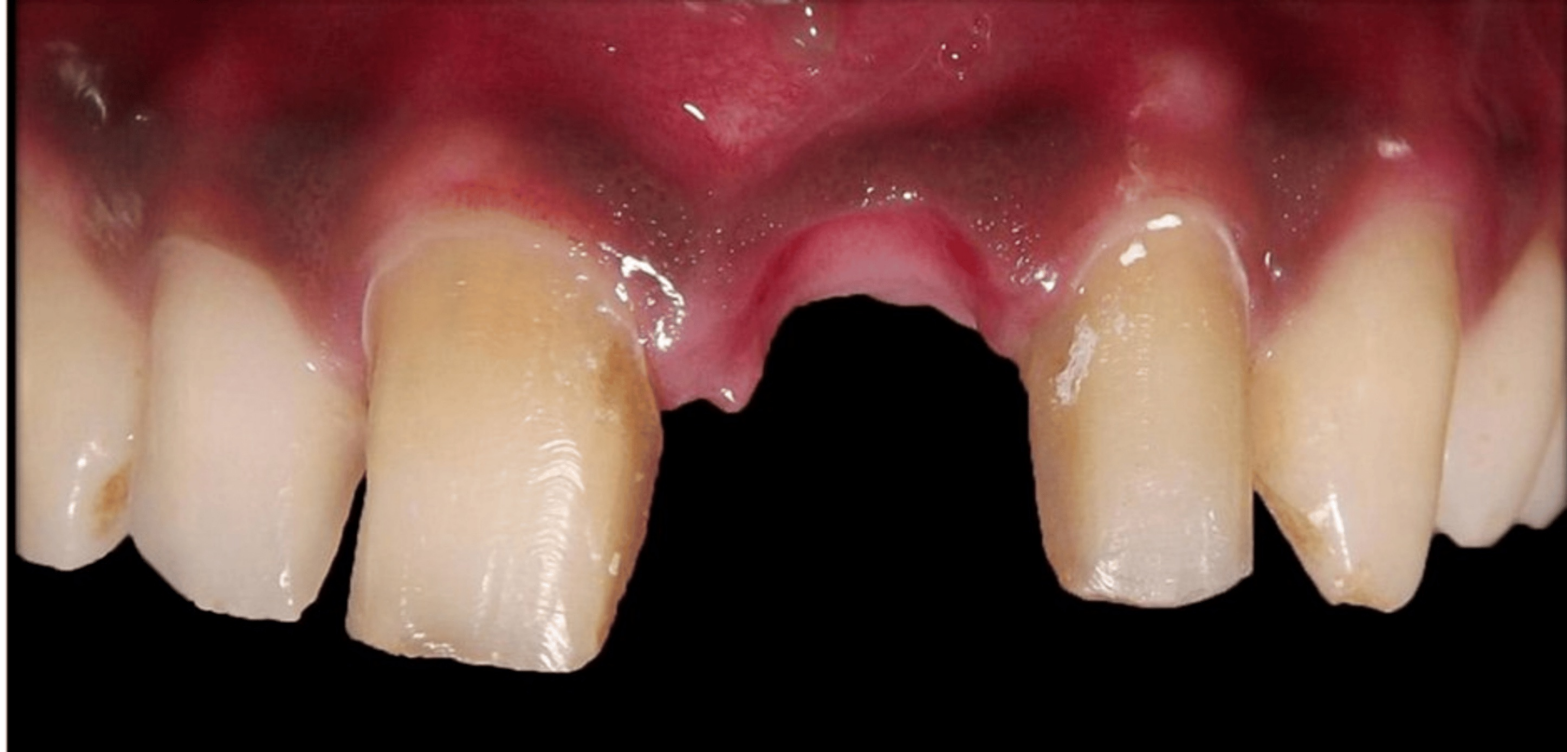
Evaluation after three weeks

To monitor tissue accommodation under the temporary pontic, the patient was recalled every seven days (Figure 5-7). To prolong tissue conditioning, a fresh coating of acrylic resin was applied after removing the temporary restoration. An examination of tissue form and aesthetics determined the amount of resin to be injected. Once the acrylic resin was added, the temporary restoration was polished and bonded. Additionally, the adapted tissue was evaluated for any signs of inflammation or plaque deposition. The fit of the provisional restoration was assessed after applying pressure. The guide for the amount of tissue to be contoured was taken from the gingival apex of the adjacent central incisor.

Final tooth preparation

**Figure 8:**
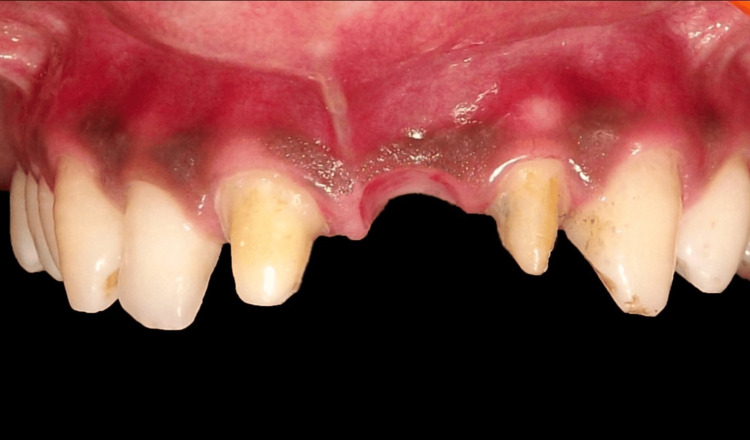
Final tooth preparation

Crown preparation was performed after the mobility of the abutments had decreased and adequate tissue depth was achieved for the pontic. A deep chamfer margin was prepared around the entire tooth, and the impression was taken using gingival retraction with retraction cords (Figure 8).

Impression technique

Because of its density, which holds the material in place while creating the impression, putty impression material was the preferred choice. The contour of the alveolus may change during impression production due to tissue breakdown caused by the removal of the interim FPD. This may result in the dental laboratory technician receiving inaccurate information about the tissue contours. To address this, tissue sculpting was performed.

Following tissue sculpting, a cast was poured using type IV dental stone to create an FPD framework, and a full-arch impression was taken. A transfer impression of the prosthesis was created using a heavy-body vinyl polysiloxane material while the temporary FPD was in place (but not secured with temporary cement). To ensure that the temporary restoration remained in position, the mouth impression was carefully removed. Petroleum jelly was applied to separate the impression from the temporary restoration.

**Figure 9:**
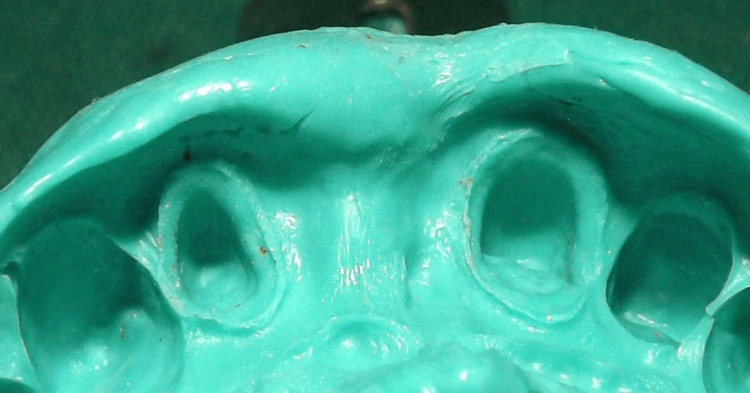
One-step putty wash impression

**Figure 10:**
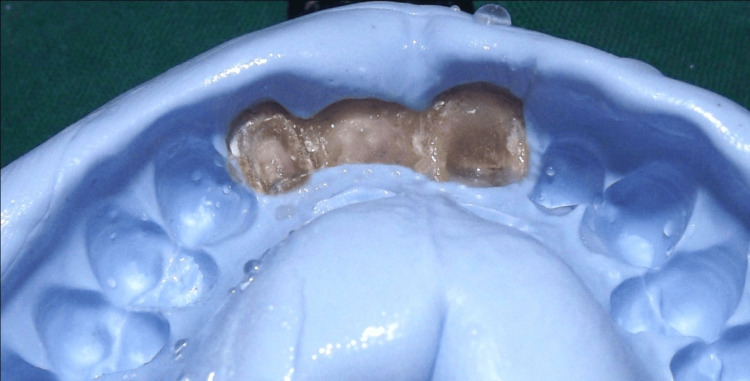
Transfer impression of putty with provisional restoration in place

**Figure 11:**
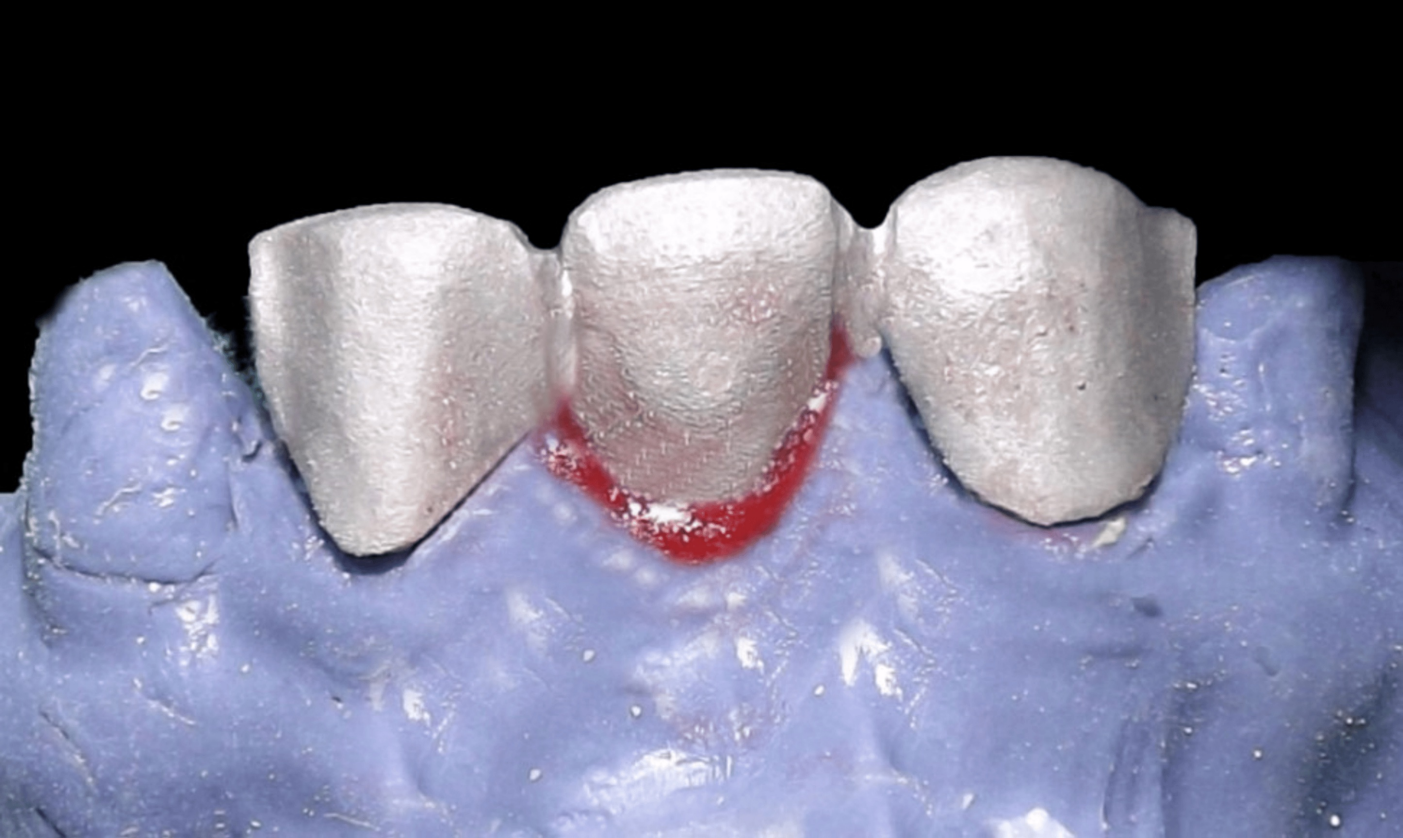
Medium-body material poured in to putty impression

To obtain a silicone cast, a medium-body elastomeric impression material was injected into the impression. After the impression material polymerized, the silicone cast was extracted. The FPD metal framework was then positioned on the silicone mold. Acrylic resin was applied underneath the pontic until it contacted the pontic site on the silicone cast. The acrylic resin replicated the gingival surface of the temporary pontic and secured it to the framework pontic (Figure 9-11).

**Figure 12:**
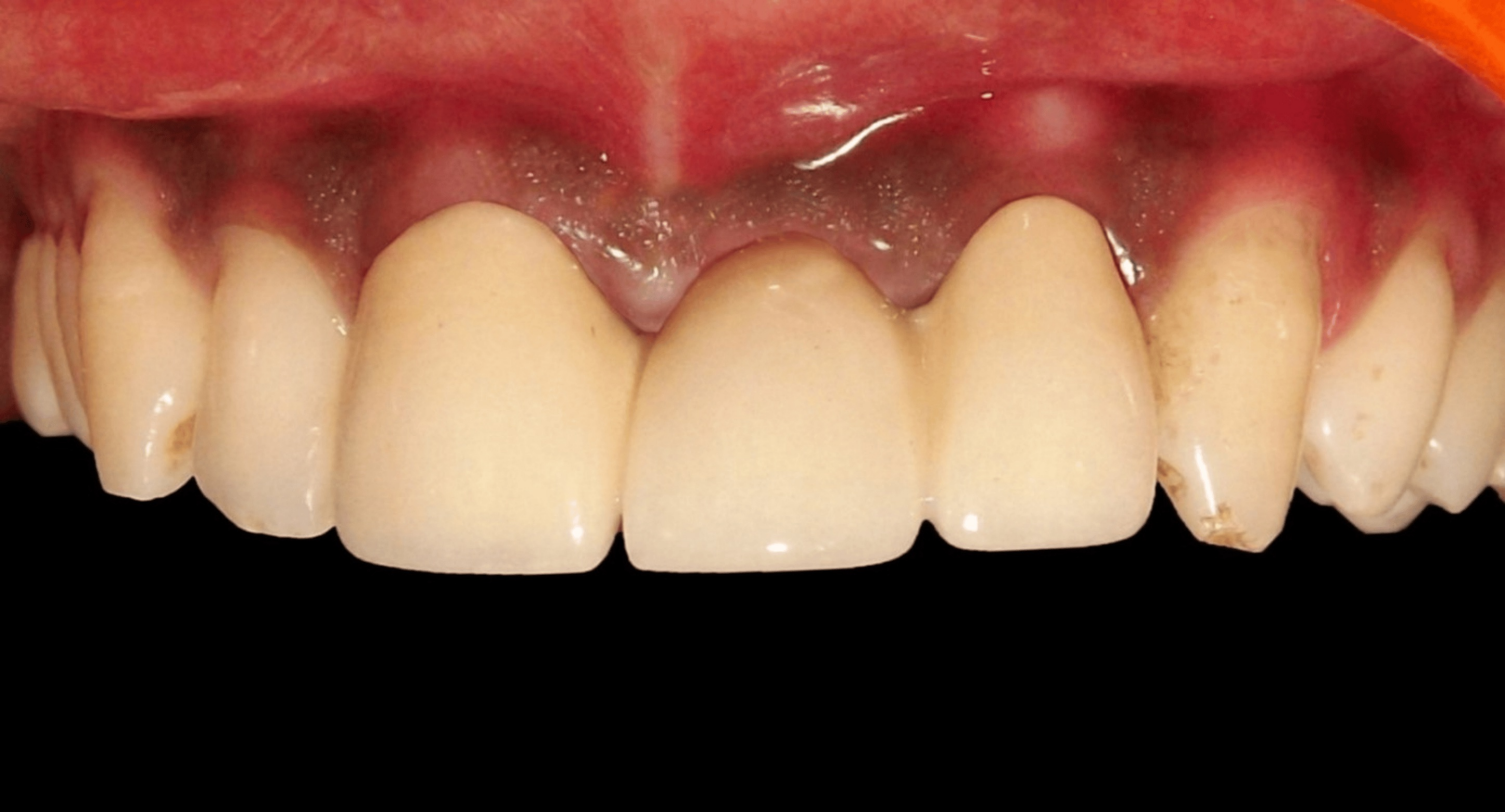
Final porcelain fused to metal prosthesis

It is important to note that the dental technician can create a pontic (Figure 12) with the exact same shape as the temporary pontic by using soft tissue cast material in the pontic site to prevent the thin plaster edges from breaking. Figure 12 shows the completed final prosthesis. Long-term follow-up was not possible, as the patient relocated from the initial place of treatment.

Table 1 presents a summary of the various stages of therapy.

**Table 1:** Summary of treatment phases

Treatment phase	Appointment number	Treatment done
Diagnostic	1	Case history recorded along with detailed clinical examination and diagnosis
Disease control	2	21 - extraction of root piece
	3	Temporization after 7 days followed by evaluation of the extraction site at 7-day intervals for the next 21 days
	4	11 and 22 - tooth preparation and putty impression recorded.
Restorative	5	Final restoration delivered
Maintenance	6	Follow up after 7 days

## Discussion

The restoration of the lost crown structure can be achieved using an implant-supported prosthesis, a three-unit FPD, or an RPD. An implant-supported prosthesis was not used as a treatment option due to the patient's preference and financial constraints. Additionally, an RPD was not selected because the patient did not comply with it.

**Figure 13:**
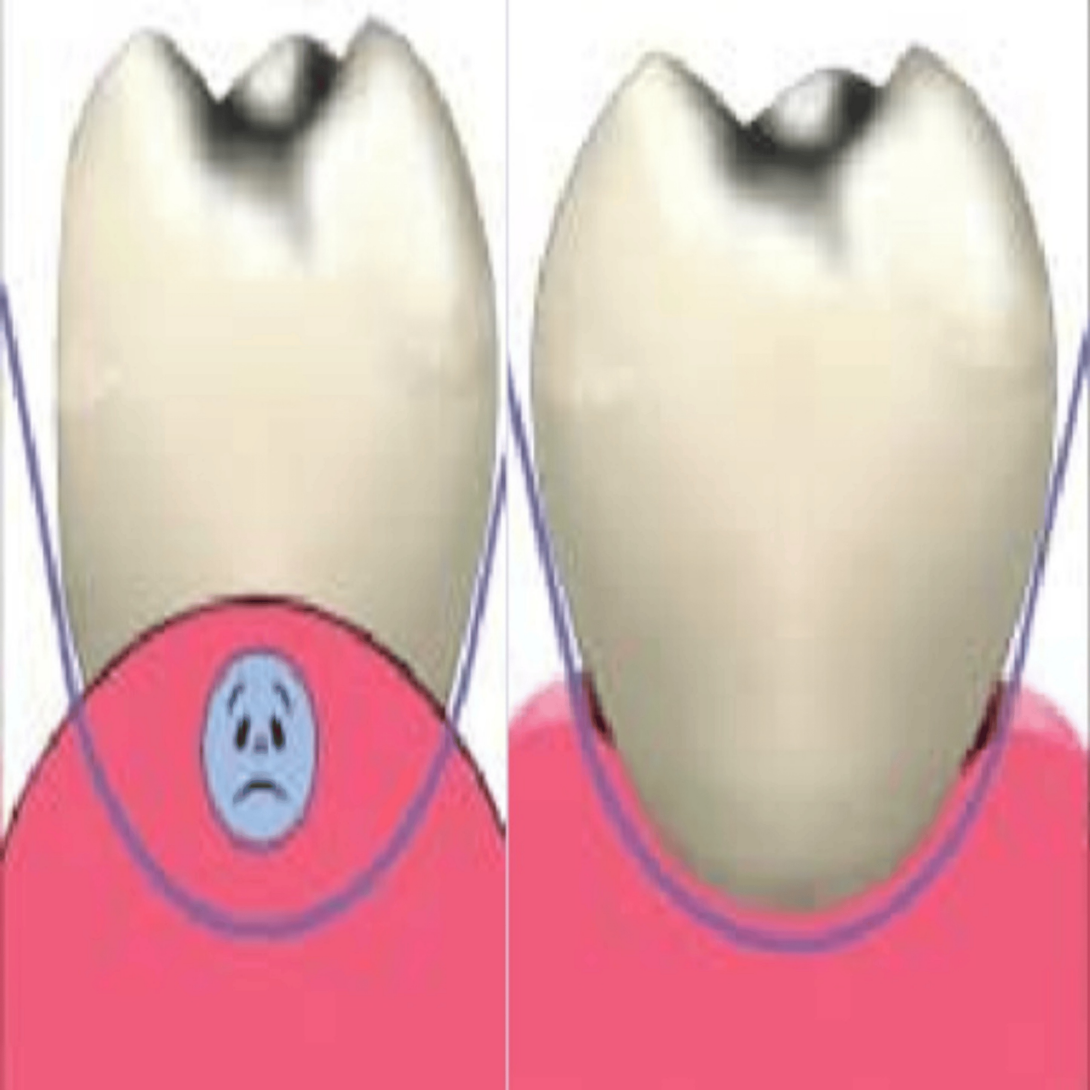
Disadvantage of modified ridge lap pontic Image Credit: Ruiz (2005) [1]. The necessary permissions have been obtained from the authors before including the image in the article.

The ovate pontic provides superior aesthetics and tissue integration, creating a natural, lifelike appearance by harmoniously blending into the alveolar ridge. It reduces food entrapment and plaque accumulation, enhancing oral hygiene and patient comfort. Compared to ridge lap and other pontic designs, it offers better long-term tissue health and stability. The ovate pontic also requires minimal tooth preparation and preserves the underlying bone structure, making it a more reliable option for both aesthetic and functional restorations. To mitigate the drawbacks of the ridge lap or modified ridge lap design, the ovate pontic was designed with a convex shape at the tissue surface rather than a concave one (Figure 13).

This makes cleaning the pontic simpler. The ovate pontic's convex shape is designed to create the proper emergence profile. However, its long-term stability may be more challenging, as the shape of the pontic may not sufficiently support occlusal forces, making it susceptible to mobility or wear.

**Figure 14:**
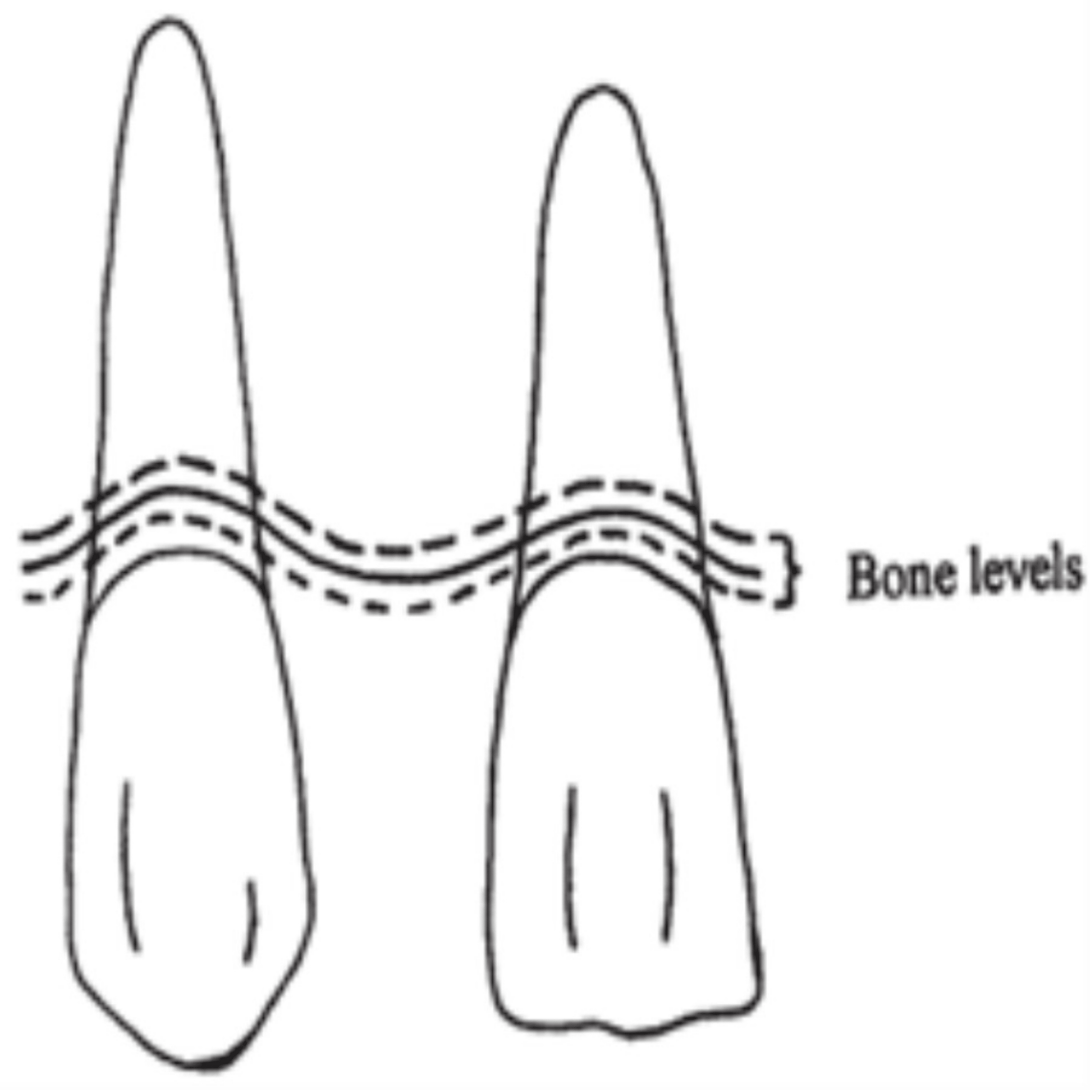
Bone crest levels Image Credit: Dylina (1999) [2]. The necessary permissions have been obtained from the authors before including the image in the article.

The dentogingival complex is a group of anatomical structures that reinforce the tooth and the overlying gum tissue, including enamel, cementum, the periodontal ligament, and gingiva. These structures are essential for tooth stability, disease prevention, and overall oral well-being. For sustained dental success, proper dentogingival complex function and health are fundamental. The dentogingival complex consists of three types of bone crests: normal bone crest, high bone crest, and low bone crest (Figure 14).

**Figure 15:**
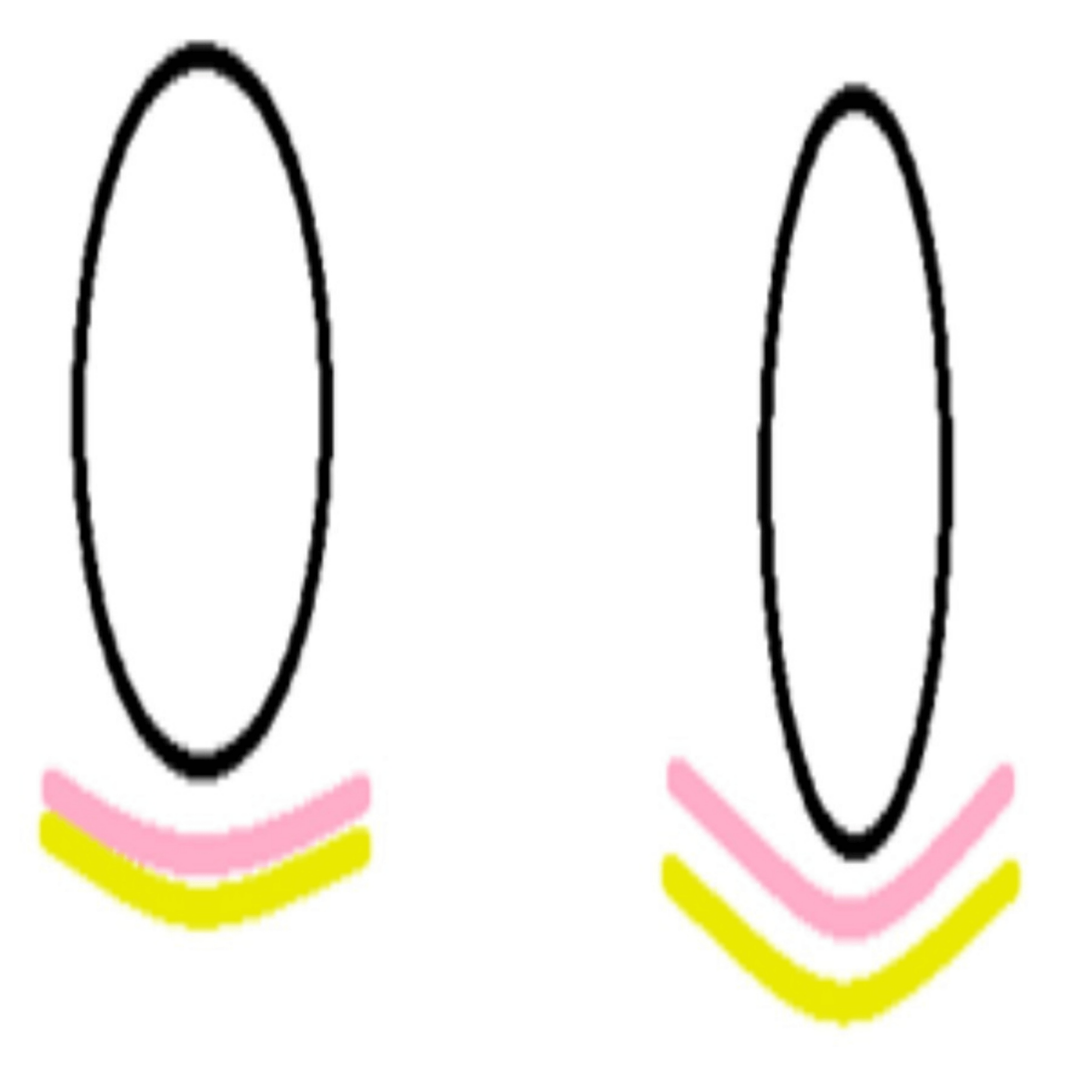
Relationship between pontic height and bone crest level Image Credit: Sumeet Agarwal

A high crest of bone allows for a shallower pontic, whereas a low crest of bone requires greater tissue support both laterally and labiolingually; therefore, the pontic should be slightly apically extended (Figure 15).

Ahmad claims that teeth have an impact on the osseous architecture and soft tissue shape within the tissue hierarchy [3]. According to Tarnow’s well-known study [4], an interdental papilla fills the gingival embrasures when the distance between the contact point and the interproximal osseous crest is 5 mm or less. Every additional 1 mm beyond 5 mm reduces the likelihood of complete fill by 50%.

Research [5] has found that the interproximal tissue and the free gingival margin on the facial bone are typically separated by 4.5 to 5.5 mm. The gingival scallop measures 3 mm in height from the crest of the alveolar bone to the free gingival edge. This means that the gingival tissue height is 1.5 to 2.5 mm higher in the interproximal area.

A pilot study was conducted to histologically evaluate the healing of gingival tissues in contact with two types of provisional ovate pontics: one made of acrylic resin with an ovate shell of low-fusing ceramic (Duceram LFC) and the other made entirely of acrylic resin (control). After two weeks of ovate pontic provisional restoration, no obvious clinical benefits were observed in three patients, as the sites treated with LFC were clinically similar to those receiving an acrylic resin pontic [6].

Researchers have described a technique for improving aesthetics by conditioning the tissue beneath the pontics through gradual displacement and pressure application [7]. Another study investigated the histological and clinical features of the human alveolar ridge mucosa adjacent to an ovate pontic-designed restoration [8]. Due to study limitations, it was concluded that there was no correlation between overt clinical indications of inflammation and the restoration of an edentulous gap with an ovate pontic, provided that appropriate oral hygiene practices were maintained. However, histologically, this pontic architecture was associated with alterations in the composition of the connective tissue compartment adjacent to the epithelium, as well as a thinner keratin layer.

## Conclusions

FPDs with ovate pontics are an excellent option for patients and dentists who prioritize aesthetics while facing economic constraints. In the present case, aesthetics and function were well maintained, and the patient was satisfied with the results. As long as they are correctly designed and maintained, ovate pontics can be used to preserve dental health, contrary to common misconceptions.

A restoration created using the transfer impression approach achieves a harmonious interaction with the surrounding soft tissues. In addition to being practical and promoting tissue health, ovate pontics offer the highest level of visual appeal. However, they require significant time and multiple appointments, which is a drawback. Other limitations include potential soft tissue irritation, patient compliance with hygiene, and the necessity of proper ridge contouring. Further research on FPD with ovate pontics is necessary.
